# Naturalistic Tobacco Retail Exposure and Smoking Outcomes in Adults Who Smoke Cigarettes Daily

**DOI:** 10.1001/jamanetworkopen.2025.30132

**Published:** 2025-09-29

**Authors:** Benjamin Muzekari, Nicole Cooper, Anthony Resnick, Alexandra M. Paul, Omaya E. Torres-Grillo, Mary E. Andrews, Bradley Mattan, Christin Scholz, Darin Johnson, José Carreras-Tartak, Melis E. Cakar, Susan Hao, Emily Zhou, Elizabeth Beard, Steven Mesquiti, Farah Sayed, Michael A. Fichman, David M. Lydon-Staley, Ian J. Barnett, Andrew A. Strasser, Thomas R. Kirchner, Lisa Henriksen, Emily B. Falk

**Affiliations:** 1 Annenberg School for Communication, University of Pennsylvania, Philadelphia; 2 Stony Brook University School of Communication and Journalism, Stony Brook, New York; 3 Alan Alda Center for Communicating Science, Stony Brook University, Stony Brook, New York; 4 Amsterdam School of Communication Research, University of Amsterdam, Amsterdam, the Netherlands; 5 School of Journalism and Mass Communications, University of South Carolina, Columbia; 6 Neuroscience Interdepartmental Program, University of California, Los Angeles; 7 Department of City and Regional Planning, Weitzman School of Design, University of Pennsylvania, Philadelphia; 8 Wharton AI & Analytics Initiative, University of Pennsylvania, Philadelphia; 9 Department of Psychology, Princeton University, Princeton, New Jersey; 10 University of Cincinnati College of Medicine, Cincinnati, Ohio; 11 Department of Bioengineering, School of Engineering and Applied Sciences, University of Pennsylvania, Philadelphia; 12 Leonard Davis Institute of Health Economics, University of Pennsylvania, Philadelphia; 13 Department of Biostatistics, Epidemiology and Informatics, Perelman School of Medicine, University of Pennsylvania, Philadelphia; 14 Center for Interdisciplinary Research on Nicotine Addiction, Department of Psychiatry, Perelman School of Medicine, University of Pennsylvania, Philadelphia; 15 Penn Medicine Abramson Cancer Center, Philadelphia, Pennsylvania; 16 Department of Social and Behavioral Sciences, School of Global Public Health, New York University, New York; 17 Center for Urban Science and Progress, Tandon School of Engineering, New York University, New York; 18 Stanford Prevention Research Center, Stanford University School of Medicine, Palo Alto, California; 19 Department of Psychology, School of Arts and Sciences, University of Pennsylvania, Philadelphia; 20 Wharton Marketing Department, University of Pennsylvania, Philadelphia; 21 Wharton Operations, Information and Decisions Department, University of Pennsylvania, Philadelphia; 22 Annenberg Public Policy Center, University of Pennsylvania, Philadelphia

## Abstract

**Importance:**

The tobacco industry spends more than $8 billion annually in the US on marketing at the point of sale. Exposure to tobacco retail has been associated with smoking outcomes, but substantially less is known about how objectively logged everyday tobacco retail exposure is associated with smoking outcomes.

**Objective:**

To assess preregistered hypotheses that individuals would report (1) greater craving and (2) more cigarettes smoked on days when their exposure to tobacco retail is higher than usual.

**Design, Setting, and Participants:**

This multimodal, within-person cohort study combined objectively logged geolocation tracking, public tobacco retail location records, and ecological momentary assessment data. Eligible participants recruited from the GeoSmoking Study were aged 21 to 65 years, smoked at least 5 cigarettes per day over the previous 6 months, owned a smartphone, and were a resident of Pennsylvania, New Jersey, or Delaware. Data were collected from May 25, 2022, to June 10, 2024.

**Exposure:**

Exposure to tobacco retail stores was assessed using mobility data matched with locations of tobacco retailers across Pennsylvania, New Jersey, and Delaware.

**Main Outcomes and Measures:**

Daily mean craving and daily number of cigarettes smoked were computed using ecological momentary assessment.

**Results:**

A total of 273 participants were included in the final analyses (mean [SD] age, 42.5 [10.7] years; 151 women [55.3%]). Multilevel models revealed support for both preregistered hypotheses. On days when individuals had more tobacco retail exposure than their own average, they reported significantly higher levels of craving (*b* = 0.04; 95% CI, 0.01-0.07; *t*_3457_ = 2.72; *P* = .01) and smoking significantly more cigarettes (*b* = 0.01; 95% CI, 0.0002-0.01; *t*_3469_ = 2.05; *P* = .04).

**Conclusions and Relevance:**

In this cohort study of individuals who smoke cigarettes daily, exposure to tobacco retail in their everyday lives was associated with increases in craving and smoking. These findings highlight the importance of retail exposure and smoking outcomes, information that is critical for developing effective tobacco control interventions and lays the foundation for broader health research on environmental factors that shape health behaviors.

## Key Points

**Question:**

Is exposure to retail marketing in people’s daily environments associated with critical health risk behaviors such as smoking?

**Findings:**

In this cohort study of 273 individuals who smoked cigarettes daily, individuals reported significantly greater cravings and smoked more cigarettes on days when their smartphone-logged tobacco retail exposure was higher than usual.

**Meaning:**

These findings suggest that tobacco retail exposure is associated with smoking dynamically across time, with important implications for public health.

## Teaser

This cohort study examines whether current-smokers’ exposure to tobacco retail marketing in their daily environments is associated with their cravings and number of cigarettes smoked.

## Introduction

Cigarette smoking is the leading cause of preventable morbidity and mortality,^1^ accounting for approximately 1 in 5 deaths in the US.^2^ Research about the daily environments that influence smoking behavior is critical to reduce this health burden. People who smoke are often exposed to tobacco advertising and products in their daily environments, as these are frequently placed near cash registers in retail outlets such as gas stations and convenience stores. These displays are commonly referred to as point-of-sale tobacco marketing or tobacco power walls.^3^ Due to advertising restrictions in other communication platforms, retail and point-of-sale marketing is the tobacco industry’s primary advertising strategy, accounting for 96.8% ($8.4 billion) of its annual marketing budget in retail environments, including at the point of sale and outdoors.^4^ What are the associations and impacts of exposure to such marketing in people’s daily lives?

Widespread exposure to tobacco retail has been linked to smoking outcomes by studies of varying designs in multiple countries.^5^ For people who smoke and recently quit smoking, tobacco retail exposure is consistently associated with increased cigarette craving, purchase urges, and impulse purchases.^6,7,8,9,10,11^ Studies that used full-size convenience store replicas or virtual stores found that reducing or eliminating visibility of retail marketing reduces purchase attempts,^12^ urges to smoke,^12^ and scores on a smoking susceptibility survey.^13^ Recent work has begun to incorporate measurement of exposure to tobacco retailers and retail marketing in individuals’ daily lives. The density of tobacco retailers in an individual’s neighborhood, a proxy for exposure, has been associated with smoking behavior.^14,15,16,17,18,19,20,21^ Using geolocation tracking, several studies have found associations between adolescents’ proximity to tobacco retailers and their smoking,^22^ as well as the success of adults’ quit attempts.^23^ This work suggests that further regulation of tobacco retail could be beneficial. However, there remains limited research that objectively quantifies naturalistic tobacco retail exposures and links those experiences with time-sensitive measures of craving and smoking, particularly in adults who smoke regularly and who are not trying to quit. Investigating these dynamics is important for understanding everyday decisions about when and how much people smoke, which may ultimately contribute to the burden of smoking-related disease.

The ubiquity of smartphones permits data collection through tools such as mobile location tracking and text messaging, enabling multimodal investigations into associations between daily life experiences and tobacco use behavior.^23^ Here, we extend prior work by integrating a combination of geolocation tracking, ecological momentary assessment,^24^ and public tobacco retail records in a preregistered^25^ study to examine time-sensitive, within-person associations among naturalistic tobacco retail exposure, craving, and smoking. We preregistered 2 confirmatory hypotheses about these associations. Hypothesis 1A posits that individuals would report higher levels of cigarette craving on days when their tobacco retail exposure is higher than usual, and hypothesis 1B posits that individuals would report higher numbers of cigarettes smoked on days when their tobacco retail exposure is higher than usual.

The temporal profile of cue-induced craving effects suggests that craving for a cigarette is a momentary experience that can fluctuate quickly and responds to many factors in an individual’s daily life (eg, mood, stress, interpersonal experiences^26,27^); therefore, craving may be associated with recent exposures.^28^ In turn, individuals may increase their smoking as a way to satisfy craving.^29^ To investigate these dynamics, we conducted exploratory analyses to examine hour-level associations between tobacco retail exposure and craving and smoking. Exploratory hypotheses included hypothesis 2A, in which we hypothesized that individuals would report higher levels of cigarette craving when their tobacco retail exposure is higher than usual in the previous hour, and hypothesis 2B, in which we hypothesized that individuals would report higher numbers of cigarettes smoked when their tobacco retail exposure is higher than usual in the previous hour.

## Methods

In this cohort study, we used data from the GeoSmoking Study, which examined associations among tobacco retail exposure, craving, smoking behavior, and neural reactivity to smoking cues.^30^ None of the variables used in the current study have been reported previously. The University of Pennsylvania Institutional Review Board approved this study. Participants provided electronic informed consent in the first online session. Methods and hypotheses were preregistered on the Open Science Framework and with ClinicalTrials.gov.^25,31^ Data were collected from May 25, 2022, to June 10, 2024. This study followed the Strengthening the Reporting of Observational Studies in Epidemiology (STROBE) reporting guideline.^32^

### Participants and Procedure

Eligible participants were aged 21 to 65 years; smoked at least 5 cigarettes per day for the past 6 months; owned an iPhone (Apple Inc) or Android OS (Google) smartphone; were residents of Pennsylvania, New Jersey, or Delaware; were fluent in English; and were fully vaccinated against COVID-19. Two days after the first online session, participants began the 14-day study period, during which geolocation tracking data were collected through Google Maps (Google), while participants completed a series of ecological momentary assessment questions each day through the RealLife Exp application (LifeData, LLC) (eFigure 1 in Supplement 1).

### Measures

Prior to beginning the study, participants could choose 1 of 4 ecological momentary assessment start times (6:00 am, 8:00 am, 10:00 am, and 12:00 pm), depending on schedule fit (eFigure 2 in Supplement 1). Craving was reported by participants using a single item, 4 times daily (Table 1). The mean value of all craving ratings for each day was calculated to measure daily craving, with higher values indicating higher levels of craving. Craving in the hour-level analyses was measured using the same single item 4 times each day.

**Table 1.** Overview of Ecological Momentary Assessment Questions^a^

Outcome and analysis	Question	Response type	Times per day	Timing category
**Craving**				
Day level, 1-h level	“Right now, how much are you craving a cigarette?”	Sliding scale from 0 = not at all to 100 = extremely in increments of 1	4	2× Signal contingent (random within a designated 4-h time bin), 2× fixed/interval contingent (ie, same time every day)
**Smoking**				
Day level	“Between [time A] and [time B], how many cigarettes have you smoked in total?”	Numeric entry	2	2× Fixed/interval contingent
1-h level	“Within the last hour, how many cigarettes did you smoke?”	Numeric entry	4	2× Signal contingent (random within a designated 4-h time bin), 2× fixed/interval contingent (ie, same time every day)

^a^ A partially random schedule was used for assessing craving and hourly smoking (2 surveys sent randomly within time bins, and 2 surveys sent at fixed times) and a fixed schedule for assessing day-level smoking (2 surveys sent at fixed times). This schedule enabled us to draw a representative sample of momentary fluctuations in craving for each day, as well as variance in recency of tobacco retail exposure to capture craving both at times when participants had been recently exposed to tobacco retailers and when they had not. Furthermore, an advantage of signal-contingent sampling is that it theoretically reduces anticipation of prompt deliveries,^33^ which may elicit more naturalistic responses. In contrast to craving, which fluctuates throughout the day, the measurement of smoking behavior may be better suited by a fixed interval, or coverage, approach. The coverage reporting approach has been shown to result in accurate, consistent responses about nicotine use, even over relatively extended periods.^34,35,36,37^

The self-reported number of cigarettes smoked daily (day-level smoking) was computed as the sum of 2 coverage assessments daily (Table 1). For missing prompts, cigarettes smoked were imputed using the participant’s average for either the first or second part of their day, corresponding to which response was missing. For our exploratory within-day analyses, the number of cigarettes smoked in the past hour (hour-level smoking) was measured using a single item 4 times each day (different from day-level smoking) collected at the same time as the craving measure.

Tobacco retail exposure was computed geospatially in relation to location logs from participants’ Google Maps data to locations of tobacco retailer licenses drawn from public records. The location of tobacco retailers is publicly available through open data portals in Pennsylvania^38^ and Delaware^39^ and by an open public records access request in New Jersey (eMethods in Supplement 1). We built a custom database containing 36 580 retailers, including 23 293 in Pennsylvania, 11 843 in New Jersey, and 1444 in Delaware. The measure captured the sum of all daily exposures to tobacco retailers (eMethods in Supplement 1).

### Statistical Analysis

To examine day-level associations between tobacco retail exposure and craving, we used a multilevel model^40^ parameterized to separate within-person and between-person associations by splitting estimators into 2 components.^41^ A time-invariant, between-person variable for tobacco retail exposure was calculated as the arithmetic mean across each participant’s repeated measures, and a time-varying, within-person estimate of tobacco retail exposure was calculated as deviations from each participant’s mean. Positive values for this within-person variable indicated days of greater levels of tobacco retail exposure compared with the individual’s average across the study period, negative values indicated days of lower levels of tobacco retail exposure compared with the individual’s average across the study period, and 0 indicated days of usual levels of tobacco retail exposure. A day-in-study variable was included to account for time as a covariate.^41^ A random intercept and slope were both included (full model details provided in the eMethods in Supplement 1). Statistical significance was evaluated at α = .05.

To examine day-level associations between tobacco retail exposure and smoking, we used an identical model to the aforementioned, substituting craving levels with cigarettes smoked. Consistent with our preregistration, all variables were winsorized within person (ie, 5th and 95th percentile cutoffs) prior to model specification to reduce the influence of outlier values. Sensitivity analyses were conducted to assess whether the covariates of age, gender (agender, genderfluid, genderqueer, man, nonbinary, woman, prefer to self-describe, prefer not to say), race (American Indian or Alaska Native, Asian, Black, Pacific Islander or Native Hawaiian, White, multiracial, prefer to self-describe, prefer not to say), ethnicity (Hispanic or Latinx, not Hispanic or Latinx, prefer not to say), smartphone type (Android OS, iOS), state of residence (Pennsylvania, New Jersey, Delaware), and nicotine dependence influence associations between tobacco retail exposure and both craving and smoking. All covariates except smartphone type were self-reported via surveys. Smartphone type was collected from Google Maps metadata.

To explore hour-level associations between tobacco retail exposure and craving, we ran 2 multilevel models,^40^ with responses nested within people. In 1 model, craving was the dependent variable and exposure during the 1-hour prior was the independent variable. In a second model, craving was the independent variable and exposure in the hour following was the dependent variable. In both models, we included time-since-start terms to account for diurnal patterns in craving and smoking. Time since start was measured by calculating the difference in minutes between the response time of the current prompt and participants’ chosen ecological momentary assessment start time selected prior to the beginning of the study.

To examine hour-level associations between tobacco retail exposure and smoking (ie, both the outcomes of exposure associated with smoking and the outcomes of smoking associated with exposure to assess reverse causation), we used identical models to those for craving, substituting craving for the hour-level smoking prompt, “Within the last hour, how many cigarettes did you smoke?” Due to the phrasing of the question, we also ran a model specifying exposures beginning 2 hours before the response time and ending 1 hour before the response time. For example, if the response time was at 8:00 pm, the ecological momentary assessment question referred to cigarettes smoked between 7:00 pm and 8:00 pm. In this additional model, exposures were measured between 6:00 pm and 7:00 pm, ensuring that all exposures were occurring prior to the period in which participants referenced their smoking count. All statistical models were computed using R, version 4.4.2 (R Foundation for Statistical Computing).

## Results

The final analytic sample included 273 participants (mean [SD] age, 42.5 [10.7] years; 3 self-identified as genderfluid or genderqueer [1.1%], 111 as men [40.7%]; 6 as nonbinary [2.2%]; and 151 as women [55.3%]; 2 self-identified as American Indian or Alaska Native [0.7%], 7 as Asian [2.6%], 64 as Black [23.4%], 175 as White [64.1%], 13 as multiracial (4.8%), and 9 as preferring not to say or to self-describe [3.2%] their race; 18 self-identified as Hispanic or Latinx [6.59%], 252 as not Hispanic or Latinx [92.31%], and 1 as preferring not to say their ethnicity). Table 2 provides additional demographics, and Table 3 provides the sample’s descriptive statistics.

**Table 2.** Sociodemographic Characteristics of 273 Participants

Characteristic	Participants, No. (%)
Age, mean (SD), y	42.5 (10.7)
Gender	
Genderfluid	2 (0.7)
Genderqueer	1 (0.4)
Man	111 (40.7)
Nonbinary	6 (2.2)
Woman	151 (55.3)
Missing	2 (0.73)
Race	
American Indian or Alaska Native	2 (0.7)
Asian	7 (2.6)
Black	64 (23.4)
White	175 (64.1)
Multiracial	13 (4.8)
Prefer not to say	1 (0.4)
Prefer to self-describe	8 (2.9)
Missing	3 (1.1)
Ethnicity	
Hispanic or Latinx	18 (6.6)
Not Hispanic or Latinx	252 (92.3)
Prefer not to say	1 (0.4)
Missing	2 (0.7)
Fagerström Test for Nicotine Dependence, mean (SD)^a^^,^^b^	5.6 (1.9)
Smartphone type	
Android OS	152 (55.7)
iOS	121 (44.3)
State of residence	
Delaware	25 (9.2)
New Jersey	43 (15.8)
Pennsylvania	205 (75.1)

^a^ Possible scores range from 0 to 10, with lower scores indicating lower nicotine dependence.

^b^ Data for 270 participants.

**Table 3.** Descriptive Statistics for 273 Participants

Characteristic	Mean (SD) [range]
Daily craving, No.	48.4 (20.4) [0-99.1]
Daily cigarettes, No.	13.5 (7.3) [3.1-75.9]
Daily geolocation observations, No.	311.3 (296.2) [6.3-2035.9]
Daily tobacco retail exposure, No.	36.7 (30.0) [1.1-206.1]
Ecological momentary assessment response rate, %	94.8 (5.2) [76.7-100]

### Preregistered Day-Level Findings

**Figure 1.**
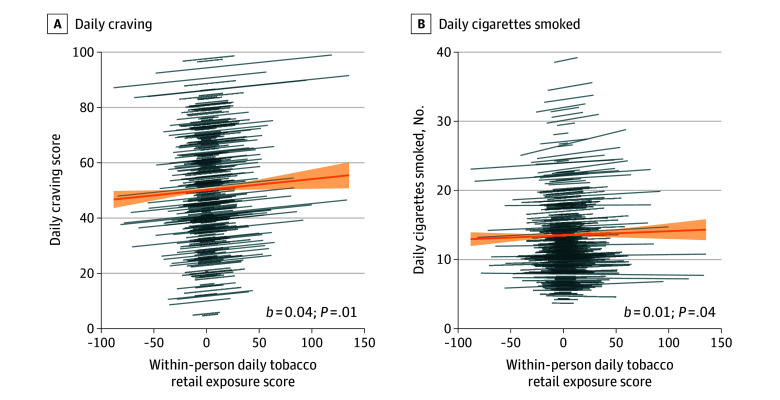
Associations Between Within-Person Daily Tobacco Retail Exposure and Craving and Smoking Displayed are the primary day-level models. The orange line and shading indicate the mean (SD) fixed effect for within-person daily tobacco retail exposure; blue lines visualize individual-level associations between within-person tobacco retail exposure and craving and daily cigarettes smoked, allowing for both random intercepts and slopes. eTables 1 to 13 in Supplement 1 provides statistics and all model specifications. One participant was excluded from this visualization to preserve the scale (B) (eMethods in Supplement 1).

As expected in hypothesis 1A, on days with higher levels of tobacco retail exposure than usual, participants reported higher levels of craving (*b* = 0.04; 95% CI, 0.01-0.07; *t*_3457_ = 2.72; *P* = .01) (Figure 1; eFigure 3 and eTables 1 and 2 in Supplement 1). All within-person associations remained significant when age, gender, race, ethnicity, state of residence, nicotine dependence, and smartphone type were included both in separate models and altogether in 1 model (eTables 6-13 in Supplement 1). As expected in hypothesis 1B, on days with greater levels of tobacco retail exposure than usual, participants reported smoking more cigarettes (*b* = 0.01; 95% CI, 0.0002-0.01; *t*_3469_ = 2.05; *P* = .04) (Figure 1; eFigure 3 and eTables 3-5 in Supplement 1). All within-person associations remained significant when including covariates both in separate models and altogether in 1 model (eTables 6-13 in Supplement 1).

### Exploratory Within-Day–Level Findings

**Figure 2.**
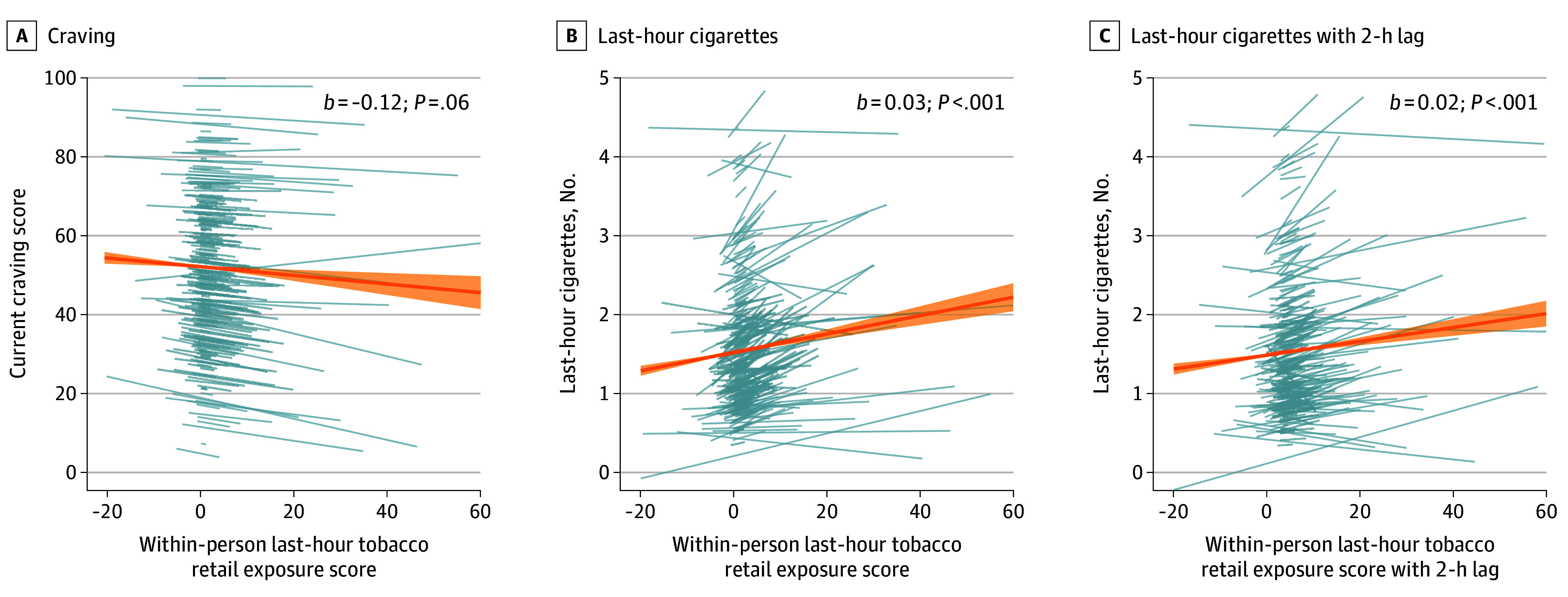
Associations Between Within-Person Hour-Level Tobacco Retail Exposure and Craving and Smoking Displayed are the hour-level model results including time since start as a covariate. The orange line and shading indicate the mean (SD) fixed effect for within-person hourly tobacco retail exposure; blue lines visualize individual-level associations between within-person tobacco retail exposure and craving and cigarettes smoked in the past hour, allowing for both random intercepts and slopes. eTables 14 to 18 in Supplement 1 provides statistics for all model specifications. One participant was excluded from this visualization to preserve the scale (B and C) (eMethods in Supplement 1).

Our primary exploratory models examined associations at the hour level, controlling for time of day (alternative models provided in the eMethods in Supplement 1). Contrary to hypothesis 2A, findings between exposure to tobacco retail in the prior hour and momentary craving were consistently negative and not statistically significant (Figure 2; eFigure 4 and eTables 14 and 15 in Supplement 1). However, as expected in hypothesis 2B, following greater levels of tobacco retail exposure than usual in the prior hour, participants consistently reported smoking significantly more cigarettes in the past hour, and this was robust to model specification (eTable 16 and 18 in Supplement 1). This association remained significant in the analysis implementing a lag to capture exposures 2 hours prior to the response time and ending 1 hour prior to the response time (*b* = 0.02; 95% CI, 0.01-0.02; *t*_13 868_ = 4.31; *P* < .001) (eTable 17 and 18 in Supplement 1).

Given that craving was directionally lower but smoking was higher during the hour prior, we examined the association between cigarettes smoked in the past hour and craving. We found that following an hour when participants smoked more than usual, they reported lower levels of craving (*b* = −10.17; 95% CI, −11.76 to −8.57; *t*_13 851_ = −12.50; *P* < .001) (eTable 19 in Supplement 1).

In the reverse models, findings between current craving and tobacco retail exposure in the following hour were consistently positive, though not always significant, suggesting that when craving was higher than usual, tobacco retail exposure was directionally higher the following hour (eTable 20 in Supplement 1). The association between cigarettes smoked in the past hour and tobacco retail exposure in the following hour was consistently positive (model including time-since-start terms: *b* = 0.11 [95% CI, 0.02-0.20; *t*_13 868_ = 2.32; *P* = .02]; model without time-since-start terms: *b* = 0.10 [95% CI, 0.01-0.19; *t*_13 871_ = 2.08; *P* = .04]), indicating that when smoking was higher than usual, tobacco retail exposure was higher the following hour (eTable 21 in Supplement 1).

## Discussion

This cohort study provides novel insight into the association between people’s exposure to different environments and their health behaviors. We examined dynamic associations between exposure to tobacco retail in people’s daily environments and cigarette smoking outcomes. We used geolocation tracking and ecological momentary assessment to investigate participants’ natural, momentary exposures and experiences across a 14-day period. We found support for both of our preregistered hypotheses, observing that participants reported higher levels of craving and smoked more cigarettes on days when they had higher levels of tobacco retail exposure than usual.

This association between daily craving and exposure to tobacco retail is consistent with and informs cue-induced craving models^42,43,44^ by suggesting that the accumulation of smoking cues correlates with higher craving in people’s natural environments across time. Prior work using approaches ranging from recalled exposure to virtual experiments has also found associations between increased exposure to tobacco retail and increased craving^9,45^ and urges to smoke.^12^ However, the naturalistic and momentary design of our approach was able to assess feelings and behaviors as they unfold while establishing individual-level exposure profiles, rather than relying on longer recall periods (which are susceptible to bias) or laboratory-based environments, which are less ecologically valid.

As a logical extension of the cue-induced craving model, we hypothesized that retail exposure would be associated with both craving and smoking behavior. People increase their smoking as a way to satisfy cravings.^29^ It is possible that if retail marketing serves as a smoking cue,^45^ it might trigger greater levels of smoking even in the absence of strong craving, which is consistent with past work that found an association between recalled exposure and neighborhood-approximated exposure to smoking outcomes.^6,17,23^ Our study adds to this literature by showing how daily environmental exposure to tobacco retail is temporally associated with craving and smoking.

Given the short temporal trajectory of cue-exposure outcomes, we investigated a finer within-day timescale and found that craving was marginally lower but that smoking was greater when participants had greater tobacco retail exposure than usual in the prior hour. We also found a negative association between current craving and smoking in the past hour. Altogether, these results suggest that participants smoked more to satisfy cravings following greater-than-usual exposure, but we did not have the temporal specificity to fully test this possibility. We then tested the reverse possibility that craving may drive exposure to retail; for example, increased craving might lead an individual to enter a store and purchase cigarettes, which would increase their tobacco retail exposure. However, the association was not robust, as significance depended on model specification.

More broadly, an important contribution of this work is the use of geolocation tracking to understand and quantify participants’ daily exposures to their environment. A common approach to studying the effects of place on naturalistic experiences is to use where participants live as an approximation of where they spend their day,^21,46^ but these methods make strong assumptions about the daily mobility of participants around their home environments. Our approach built on several initial studies using geolocation to explore exposure to tobacco retail.^22,23^ Future work could also incorporate qualitative contextualization for geolocation and survey data, such as interview methods, to gain multifaceted insight into how place-based experiences could be associated with smoking behaviors.^47^ Our findings bolster the importance of a dynamic conception of risk, showing that across days and even within days, smoking levels are associated dynamically with tobacco retail exposure. This approach provides a strong foundation for expansion in future health behavior research.

The widespread adoption of sensing technology, such as smartphones^48^ and wearable devices,^49^ could enable refinement of theoretical models of health behavior, such as cue-induced craving, in daily life. Combining geolocation tracking with time-intensive methods such as ecological momentary assessment^50,51,52,53^ (often termed geographically explicit ecological momentary assessment) could also make it feasible to obtain rich data about how other daily environmental exposures, such as other forms of retail exposure (eg, alcohol), poverty exposure,^54^ and weather events,^55^ may be associated with substance use, including through the elicitation of antecedents such as negative affect and stress.^27,56,57^ The dynamic assessment of such environmental risks is particularly relevant for just-in-time interventions,^58,59^ a health behavior change approach that provides support at a critical moment and in a particular context in which the individual most needs it.^60^

### Limitations

Our findings should be considered in the context of the study’s limitations. The design was correlational, and despite our fine-grained temporal analyses, further research is needed to show causal effects of tobacco retail exposure in an individual’s everyday environment. Another limitation is that the operationalization of exposure to risk factors in individuals’ daily environments, including tobacco retail environments and marketing, does not yet have clear standards (eg, temporally, spatially).^61,62,63,64^ Our method is also restricted in being able to distinguish between indoor vs outdoor marketing or whether the individual physically entered the retailer. As the health sciences continue to integrate information about everyday human mobility and develop geospatial analysis methods, future research is needed to understand the most appropriate ways to model environmental exposures.

## Conclusions

This cohort study found that on days when participants had greater levels of retail exposure than usual, they reported both higher levels of craving and smoking more cigarettes. This study provides insight into dynamic associations between naturalistic exposure to tobacco retailers and smoking behaviors, which may inform public policy and health behavior change interventions to reduce smoking. More broadly, this work may also lay the foundation for the broader combination of geolocation, ecological momentary assessment, and publicly available data to quantify the risk and protective factors in people’s daily environments and link these to their daily experiences, behaviors, and health outcomes.

## Notes

Supplement 1.
eMethods.

eReferences.
**eFigure 1.** Timeline for Study Participants **eFigure 2.** EMA Survey Schedule Clock Example **eFigure 3.** Associations Between Within-Person Daily Tobacco Retail Exposure and Craving and Smoking, Including Smoking Outlier **eFigure 4.** Associations Between Within-Person Hour-Level Tobacco Retail Exposure and Craving and Smoking, Including Smoking Outlier **eTable 1.** Daily Craving (Winsorized): Day Level **eTable 2.** Daily Craving (Nonwinsorized): Day Level **eTable 3.** Daily Cigarettes (Winsorized): Day Level **eTable 4.** Daily Cigarettes (Nonwinsorized): Day Level **eTable 5.** Daily Cigarettes (Excluding Smoking Outlier 8.46 SD Above the Mean): Day Level **eTable 6.** Daily Craving and Cigarettes (Age as a Covariate): Day Level **eTable 7.** Daily Craving and Cigarettes (Gender as a Covariate): Day Level **eTable 8.** Daily Craving and Cigarettes (Race as a Covariate): Day Level **eTable 9.** Daily Craving and Cigarettes (Ethnicity as a Covariate): Day Level **eTable 10.** Daily Craving and Cigarettes (Fagerström Test for Nicotine Dependence as a Covariate): Day Level **eTable 11.** Daily Craving and Cigarettes (Smartphone Type as a Covariate): Day Level **eTable 12.** Daily Craving and Cigarettes (State as a Covariate): Day Level **eTable 13.** Daily Craving and Cigarettes (All Covariates Included): Day Level **eTable 14.** Current Craving: Hour Level **eTable 15.** Current Craving (Last-Hour Cigarettes as a Covariate): Hour Level **eTable 16.** Last-Hour Cigarettes: Hour Level **eTable 17.** Last-Hour Cigarettes (2-Hour Exposure Lag): Hour Level **eTable 18.** Last-Hour Cigarettes (Excluding Smoking Outlier 8.18 SD Above the Mean): Hour Level (1-Hour and 2-Hour Exposure Lag) **eTable 19.** Craving and Smoking Association: Hour Level **eTable 20.** Current Craving and Next-Hour Exposure: Hour-Level Reverse **eTable 21.** Last-Hour Cigarettes and Next-Hour Exposure: Hour-Level Reverse

Supplement 2.
Data Sharing Statement
