# Supplementary material for: Naturalistic Tobacco Retail Exposure and Smoking Outcomes in Adults Who Smoke Cigarettes Daily
Source: JAMA Netw Open. 2025 Sep 29;8(9):e2530132. doi: 10.1001/jamanetworkopen.2025.30132 (PMC12481226; doi:10.1001/jamanetworkopen.2025.30132)
Supplement: Supplement 1. — eMethods. eReferences. eFigure 1. Timeline for Study Participants eFigure 2. EMA Survey Schedule Clock Example eFigure 3. Associations Between Within-Person Daily Tobacco Retail Exposure and Craving and Smoking, Including Smoking Outlier eFigure 4. Associations Between Within-Person Hour-Level Tobacco Retail Exposure and Craving and Smoking, Including Smoking Outlier eTable 1. Daily Craving (Winsorized): Day Level eTable 2. Daily Craving (Nonwinsorized): Day Level eTable 3. Daily Cigarettes (Winsorized): Day Level eTable 4. Daily Cigarettes (Nonwinsorized): Day Level eTable 5. Daily Cigarettes (Excluding Smoking Outlier 8.46 SD Above the Mean): Day Level eTable 6. Daily Craving and Cigarettes (Age as a Covariate): Day Level eTable 7. Daily Craving and Cigarettes (Gender as a Covariate): Day Level eTable 8. Daily Craving and Cigarettes (Race as a Covariate): Day Level eTable 9. Daily Craving and Cigarettes (Ethnicity as a Covariate): Day Level eTable 10. Daily Craving and Cigarettes (Fagerström Test for Nicotine Dependence as a Covariate): Day Level eTable 11. Daily Craving and Cigarettes (Smartphone Type as a Covariate): Day Level eTable 12. Daily Craving and Cigarettes (State as a Covariate): Day Level eTable 13. Daily Craving and Cigarettes (All Covariates Included): Day Level eTable 14. Current Craving: Hour Level eTable 15. Current Craving (Last-Hour Cigarettes as a Covariate): Hour Level eTable 16. Last-Hour Cigarettes: Hour Level eTable 17. Last-Hour Cigarettes (2-Hour Exposure Lag): Hour Level eTable 18. Last-Hour Cigarettes (Excluding Smoking Outlier 8.18 SD Above the Mean): Hour Level (1-Hour and 2-Hour Exposure Lag) eTable 19. Craving and Smoking Association: Hour Level eTable 20. Current Craving and Next-Hour Exposure: Hour-Level Reverse eTable 21. Last-Hour Cigarettes and Next-Hour Exposure: Hour-Level Reverse [file jamanetwopen-e2530132-s001.pdf]

Supplemental Online Content

Muzekari B, Cooper N, Resnick A, et al. Naturalistic tobacco retail exposure and smoking outcomes in adults who smoke cigarettes daily. *JAMA Netw Open*. 2025;8(9):e2530132. doi:10.1001/jamanetworkopen.2025.30132

eMethods

eReferences

eFigure 1. Timeline for Study Participants

eFigure 2. EMA Survey Schedule Clock Example

eFigure 3. Associations Between Within-Person Daily Tobacco Retail Exposure and Craving and Smoking, Including Smoking Outlier

eFigure 4. Associations Between Within-Person Hour-Level Tobacco Retail Exposure and Craving and Smoking, Including Smoking Outlier

eTable 1. Daily Craving (Winsorized): Day Level

eTable 2. Daily Craving (Nonwinsorized): Day Level

eTable 3. Daily Cigarettes (Winsorized): Day Level

eTable 4. Daily Cigarettes (Nonwinsorized): Day Level

eTable 5. Daily Cigarettes (Excluding Smoking Outlier 8.46 SD Above the Mean): Day Level

eTable 6. Daily Craving and Cigarettes (Age as a Covariate): Day Level

eTable 7. Daily Craving and Cigarettes (Gender as a Covariate): Day Level

eTable 8. Daily Craving and Cigarettes (Race as a Covariate): Day Level

eTable 9. Daily Craving and Cigarettes (Ethnicity as a Covariate): Day Level

eTable 10. Daily Craving and Cigarettes (Fagerström Test for Nicotine Dependence as a Covariate): Day Level

eTable 11. Daily Craving and Cigarettes (Smartphone Type as a Covariate): Day Level

eTable 12. Daily Craving and Cigarettes (State as a Covariate): Day Level

eTable 13. Daily Craving and Cigarettes (All Covariates Included): Day Level

eTable 14. Current Craving: Hour Level

eTable 15. Current Craving (Last-Hour Cigarettes as a Covariate): Hour Level

eTable 16. Last-Hour Cigarettes: Hour Level

eTable 17. Last-Hour Cigarettes (2-Hour Exposure Lag): Hour Level

eTable 18. Last-Hour Cigarettes (Excluding Smoking Outlier 8.18 SD Above the Mean): Hour Level (1-Hour and 2-Hour Exposure Lag)

© 2025 Muzekari B et al. *JAMA Network Open*.

eTable 19. Craving and Smoking Association: Hour Level
eTable 20. Current Craving and Next-Hour Exposure: Hour-Level Reverse
eTable 21. Last-Hour Cigarettes And Next-Hour Exposure: Hour-Level Reverse

This supplemental material has been provided by the authors to give readers additional information about their work.

© 2025 Muzekari B et al. *JAMA Network Open*.

eMethods

Procedure

Recruitment materials were distributed by BuildClinical, who advertised the study on digital platforms such as Google, Facebook, and WebMD. All advertisements included a link directing interested participants to complete an initial screening survey. Survey respondents who were potentially eligible to participate were invited to sign up for an initial call to learn more about the study and to confirm eligibility criteria. After the initial call, eligible participants were directed to a screening, which began with an electronic consent form. Participants then provided their shipping address and submitted a picture of their COVID-19 vaccination card. The instructions guided them through downloading and/or setting up Google Maps on their phone, then exporting and uploading their Google timeline (location history) data. Potential participants could complete this step while on the phone/video call with the experimenter or at a later, self-guided time. Experimenters then confirmed A) that potential participants lived in the study area (Pennsylvania (PA), New Jersey (NJ), or Delaware (DE)) using the uploaded timeline data and shipping address and B) that the potential participant was fully vaccinated against COVID-19. Eligible individuals were then mailed a box containing study materials, including a urine cotinine test, KN95/KF94 or N95 masks, and a Greenphire Clincard. Upon receipt of the study materials, participants were given instructions to enroll in the study and begin their participation by completing the first online session, which began with an electronic consent form. Additional protocol details relevant to Strengthening the Reporting of Observational Studies in Epidemiology (STROBE) guidelines can be found in a separate protocol paper[1].

Eligibility Criteria

Eligibility criteria are described in a separate, protocol paper[1] as: “Individuals were eligible to participate if they were between the ages of 21-65; smoked at least 5 cigarettes a day for the past 6 months; owned an iPhone or Android smartphone that could be used on a daily basis; were residents of Pennsylvania, New Jersey, or Delaware; were fluent in English; and were fully vaccinated against COVID-19. Individuals were excluded during the screening process if they did not meet the eligibility criteria above or for meeting one or more of the following exclusion criteria: concurrent enrollment or plans to enroll in a smoking cessation program within 3 months of completing the screening survey; plans to use nicotine substitutes or smoking cessation treatments within 3 months of completing the screening survey; demonstration of urine cotinine concentration below 200 ng/mL (≥ 200 ng/mL confirmed smoking status); pregnancy; refusal to install Google Maps or LifeData applications on their mobile phone; their phone’s functionality did not allow for adequate completion of study tasks; inability or refusal to upload Google Timeline data after receiving instructions and any additional guidance; any planned, extended trips outside of PA, NJ, and DE during the study phase (participants were also given the option to postpone enrollment).”

Additional Measures Information

Tobacco Retail Exposure. Participants accessed their geolocation history through Google Takeout and uploaded their data to a study website. Each location observation was tagged with latitude, longitude, and a timestamp.

The study team downloaded PA and DE retailer lists monthly. The NJ list was updated by the state, and requested by the study team, yearly. The tobacco retailer lists contain retail store names, license numbers, street addresses, and license start and end dates. PA and DE datasets typically contained latitude and longitude information. When latitude and longitude information were missing, or in the case of NJ which did not provide latitude and longitude information, addresses were geocoded using the Google Places Application Programming Interface.

To reduce false negatives due to variability in the accuracy of retailer locations and occasional sparsity of participant location points, we used a multistep algorithm to assess whether each location point would be counted as an exposure to a tobacco retailer. If a given point was within 100 feet of a tobacco retailer, that point was counted as an exposure. Two or more consecutive points within 1,000 feet of a tobacco retailer were also counted as an exposure. All consecutive points within 100 or 1,000 feet of a retailer were counted as a single exposure. If a participant was within the specified distance from multiple retailers at the same time, multiple exposures were counted.

Data Preparation and Analysis

© 2025 Muzekari B et al. *JAMA Network Open*.

To examine day-level associations between tobacco retail exposure and craving, we used a multilevel model[2] parameterized to separate within-person and between-person associations by splitting predictors into time-invariant (between-person) and time-varying (within-person) components[3]. A time-invariant, between-person variable for tobacco retail exposure was calculated as the arithmetic mean across each participant's repeated measures and a time-varying, within-person estimate of tobacco retail exposure was calculated as deviations from each participant's mean. At level 1 (day-level variables), the formal model was constructed as:

$$\boldsymbol{Craving_{it} = \beta_{0i} + \beta_{1i} TobaccoRetailExposure\_within_{it} + \beta_{2i}\, DayOfStudy_{it} + e_{it}}$$

where $Craving_{it}$ is the average craving for person i on day t; β_{0i} indicates the expected craving on a typical day for the prototypical person in the sample; β_{1i} indicates the fixed effect of within-person differences in tobacco retail exposure on craving, relative to the person's own average; β_{2i} indicates the effect of day in study on craving to account for time as a covariate[3]; and e_{it} are day-specific residuals that are allowed to autocorrelate. Person-specific intercepts and associations (from the Level 1 model) were specified (at Level 2) as:

$$\boldsymbol{\beta_{0i} = \gamma_{00} + \gamma_{01} TobaccoRetailExposure\_average_i + u_{0i}}$$
$$\boldsymbol{\beta_{1i} = \gamma_{10} + u_{1i}}$$
$$\boldsymbol{\beta_{2i} = \gamma_{20}}$$

where the γs are sample-level parameters and u_{0i} is a residual between-person difference that is uncorrelated with e_{it}.

To examine day-level associations between tobacco retail exposure and smoking, we used an identical model to the above, substituting craving levels with cigarettes smoked.

To examine exploratory hour-level associations between retail exposure and craving, we subset the geolocation tracking data to focus on tobacco retail exposure during the one hour prior to the participant's craving prompt entry for exposure → craving analyses (or the one hour following the craving prompt for the craving → exposure analyses). Prompt-craving was measured using the same ecological momentary assessment (EMA) item from the day-level analysis. Again, we used a multilevel model[2], but measured tobacco retail exposure in the one hour prior to the response time rather than across the entire day. We also included time-since-start to account for diurnal patterns in craving and smoking. Time-since-start was measured by calculating the difference in minutes between the response time of the current prompt and participants' chosen EMA start-time selected prior to the beginning of the study.

At level 1 (prompt-level variables), the formal model was constructed as:

$$\boldsymbol{Craving_{it} = \beta_{0i} + \beta_{1i} PriorHourTobaccoRetailExposure\_within_{it} + \beta_{2i}\, DayOfStudy_{it} + \beta_{3i}\, TimeSinceStartLinear_{it} + \beta_{4i} TimeSinceStartQuadratic_{it} + \beta_{5i} TimeSinceStartCubic_{it} + e_{it}}$$

where $Craving_{it}$ is the craving reported by person i at prompt t; β_{0i} indicates the expected craving at a typical prompt for the prototypical person in the sample; β_{1i} indicates the fixed effect of within-person differences in prior hour's tobacco retail exposure on craving, relative to the person's own average; β_{2i} indicates the effect of day in study on craving to account for time as a covariate[3]; β_{3i}, β_{4i}, and β_{5i} indicate the effects of time-since-start on craving to account for diurnal patterns as a covariate, and e_{it} are prompt-specific residuals that are not allowed to autocorrelate due unequal spacing between sequential measurements. Person-specific intercepts and associations (from the Level 1 model) were specified (at Level 2) as:

$$\boldsymbol{\beta_{0i} = \gamma_{00} + \gamma_{01} PriorHourTobaccoRetailExposure\_average_i + u_{0i}}$$
$$\boldsymbol{\beta_{1i} = \gamma_{10} + u_{1i}}$$
$$\boldsymbol{\beta_{2i} = \gamma_{20}}$$
$$\boldsymbol{\beta_{3i} = \gamma_{30}}$$

where the γs are sample-level parameters and u_{0i} is a residual between-person difference that is uncorrelated with e_{it}.

For analyses exploring the potential impact of craving on exposure, we parameterized the model in reverse, with tobacco retail exposure in the hour following the prompt as the outcome. At level 1 (prompt-level variables), the formal model was constructed as:

$$\boldsymbol{TobaccoRetailExposure_{it} = \beta_{0i} + \beta_{1i} Craving\_within_{it} + \beta_{2i}\, DayOfStudy_{it} + \beta_{3i}\, TimeSinceStartLinear_{it} + \beta_{4i} TimeSinceStartQuadratic_{it} + \beta_{5i} TimeSinceStartCubic_{it} + e_{it}}$$

and person-specific intercepts and associations (from the Level 1 model) were specified (at Level 2) as:

$$\boldsymbol{\beta_{0i} = \gamma_{00} + \gamma_{01} Craving\_average_i + u_{0i}}$$

© 2025 Muzekari B et al. *JAMA Network Open*.

$\beta_{1i} = \gamma_{10} + u_{1i}$
$\beta_{2i} = \gamma_{20}$
$\beta_{3i} = \gamma_{30}$

where the γs are sample-level parameters and u_{0i} is a residual between-person difference that is uncorrelated with e_{it}.

To examine these hour-level associations between retail exposure and smoking (i.e. both the impact of exposure on smoking and the impact of smoking on exposure), we used identical models above substituting craving for hour-level smoking prompt (i.e. "Within the last hour, how many cigarettes did you smoke?"). Due to the phrasing of the question, we also ran a model specifying exposures beginning two hours before the response time and ending one hour before the response time. For example, if the response time was at 8 PM, the EMA question refers to cigarettes smoked between 7 PM and 8 PM. In this additional model, exposures are measured between 6 PM and 7 PM, ensuring that all exposures are occurring prior to the period in which participants reference their smoking count.

All statistical models were computed using the R (version 4.4.2) software[4]. Models were computed using the nlme package[5]. Primary day-level models were first fitted using maximum likelihood both allowing and not allowing for autocorrelation. Akaike information criterion, Bayesian information criterion, and log-likelihood ratio metrics showed that both craving and smoking models had a greater fit when allowing for autocorrelation among the residuals. All final models, both day-level and hour-level, were fit using restricted maximum likelihood. Hour-level models did not allow for autocorrelation and included linear, quadratic, and cubic terms for the time-since-start variable. Both quadratic and cubic terms were significantly associated with craving and smoking in the base models, and therefore were incorporated throughout exploratory models that included time-since-start as a covariate.

Excluded Participants

Of the 310 participants enrolled, 37 were excluded prior to the final analysis. Participants were excluded for low response rate (responded to less than 75% of the EMA prompts; n=13), technical issues (n=10), lost contact (n=5), incompatible data uploaded (n=4), withdrew after consent (n=3), and ineligibility due to age (n=1) and failed cotinine test (n=1). The final analysis sample in this paper (n=273) accounts for specific data quality exclusion criteria that are unique to this analysis pipeline and therefore reports a lower sample size than the protocol paper for this study's baseline period[1].

To test for robustness in day-level models, we excluded one participant with an extremely high cigarette count (8.46 SD above the mean) in an alternative model specification (eTable 5). Within-person associations remained significant. To test for robustness in hour-level models, we excluded the same participant with an extremely high cigarette count (8.18 SD above the mean) in alternative model specifications (eTable 18). Within-person associations remained significant.

Descriptive Statistics

Statistical tests were performed to assess associations between data missingness and covariates. Kruskal-Wallis tests revealed no significant associations between the number of days with EMA data and gender, ethnicity, race, and state of residence (all p values >0.05). A Kruskal-Wallis test revealed a significant association between the number of days with EMA data and operating system ($\chi2(1)=8.77$, $p=0.003$). Android users (M=13.89) had significantly more days with EMA data than IOS users (M=13.51). Linear regression tests revealed no significant associations between the number of days with EMA data and age and Fagerström Test for Nicotine Dependence scores (both p values >0.05). We included all covariates in robustness checks.

Statistical tests were also performed to assess associations between average daily geolocation observations and covariates. Kruskal-Wallis tests revealed no significant associations between average daily geolocation observations and ethnicity, race, and state of residence (all p values >0.05). A Kruskal-Wallis test revealed a significant association between average daily geolocation observations and gender ($\chi2(4)=15.97$, $p=0.003$). A post-hoc analysis revealed men had a significantly greater amount of average daily geolocation observations (M=366.72) than women (M=272.47; p=0.01). A Kruskal-Wallis test revealed a significant association between average daily geolocation observations and operating system ($\chi2(1)=183.94$, $p<0.001$). Android users (M=493.02) had significantly more average daily geolocation observations than IOS users (M=83.09). Linear regression tests revealed no significant associations between average daily geolocation observations and age and Fagerström Test for Nicotine Dependence scores (both p values >0.05).

Citation Diversity Statement

© 2025 Muzekari B et al. *JAMA Network Open*.

Recent work in several fields of science has identified a bias in citation practices such that papers from women and other minority scholars are under-cited relative to the number of such papers in the field[6,7]. Here we present citation statistics in the interest of increasing transparency. First, we obtained the predicted gender of the first and last author of each reference by using databases that store the probability of a first name being carried by a woman[8,9]. By this measure and excluding self-citations to the first and last authors of our current paper), our references contain 28.96% woman(first)/woman(last), 12.7% man/woman, 21.67% woman/man, and 36.67% man/man. This method is limited in that a) names, pronouns, and social media profiles used to construct the databases may not, in every case, be indicative of gender identity and b) it cannot account for intersex, non-binary, or transgender people. We look forward to future work that could help us to better understand how to support equitable practices in science.

eReferences

1. Cooper N, Muzekari B, Andrews M, et al. Using ecological momentary assessment, geolocation tracking, and neuroimaging to assess effects of tobacco retail exposure on smoking behavior: protocol for the GeoSmoking Study. Published online February 28, 2025. https://doi.org/10.31234/osf.io/bgkze_v2

2. Snijders TAB, Bosker RJ. *Multilevel Analysis: An Introduction to Basic and Advanced Multilevel Modeling*. 2nd ed. Sage; 2012.

3. Bolger N, Laurenceau JP. *Intensive Longitudinal Methods: An Introduction to Diary and Experience Sampling Research*. Guilford Press; 2013.

4. R Core Team. R: A Language and Environment for Statistical Computing. Published online 2024. https://www.R-project.org/

5. José Pinheiro, Douglas Bates, R Core Team. nlme: Linear and Nonlinear Mixed Effects Models. Published online 2024. https://CRAN.R-project.org/package=nlme

6. Wang X, Dworkin JD, Zhou D, et al. Gendered citation practices in the field of communication. Ann Int Commun Assoc. 2021;45(2):134-153. doi:10.1080/23808985.2021.1960180

7. Chatterjee P, Werner RM. Gender Disparity in Citations in High-Impact Journal Articles. JAMA Netw Open. 2021;4(7):e2114509. doi:10.1001/jamanetworkopen.2021.14509

8. Dworkin JD, Linn KA, Teich EG, Zurn P, Shinohara RT, Bassett DS. The extent and drivers of gender imbalance in neuroscience reference lists. Nat Neurosci. 2020;23(8):918-926. doi:10.1038/s41593-020-0658-y

9. Zhou D, Bertolero M, Stiso J, et al. Gender diversity statement and code notebook v1.1.1. Published online January 14, 2022. doi:10.5281/zenodo.4104748

© 2025 Muzekari B et al. *JAMA Network Open*.

eFigure 1. Timeline for Study Participants. Those who expressed interest in the study were directed to Online Screening Survey A. Survey respondents who were potentially eligible based on Online Screening Survey A responses were invited to sign up for an initial call to learn more about the study and to confirm eligibility criteria. After the initial call, participants were directed to Online Screen B, which asked additional questions to determine eligibility criteria. Eligible participants were then mailed a box containing study materials, including a urine cotinine test, KN95/KF94 or N95 masks, and a Greenphire Clincard. After receipt, participants provided electronic consent to enroll in the study and proceeded to complete Online Survey Session #1. During this session, participants followed the provided instructions to complete a urine cotinine test and self-report measures; received instructions for the Baseline period EMA task and answered questions assessing their comprehension of the task; and installed, set up, and practiced using RealLife Exp (the EMA app) on their smartphone. Two days after completing this session, participants completed the Baseline period of the study. This period lasted 14 days, during which geolocation tracking was enabled through Google Maps, while participants completed a series of EMA questions each day through the RealLife Exp app. After completion of the Baseline period, participants uploaded their Google Timeline location history records and completed a battery of questionnaires.

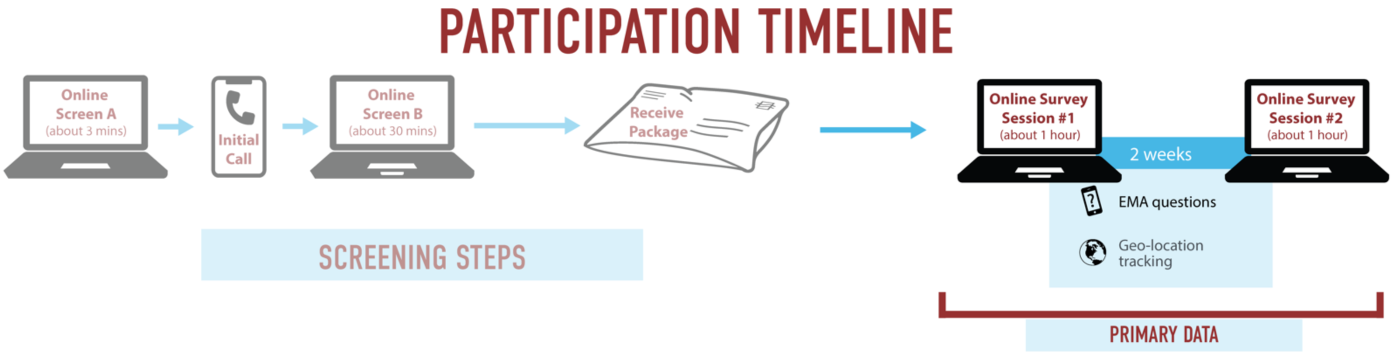

© 2025 Muzekari B et al. *JAMA Network Open*.

eFigure 2. EMA Survey Schedule Clock Example. Displayed is an EMA survey schedule clock example for a participant with an 8am start-time. Start-times varied across participants and were self-selected based on schedule fit. Craving prompt 1 is sent at a random time between 8am and 12 pm. Craving prompt 2 and Smoking prompt 1 are sent at a fixed time at 1pm. Craving prompt 3 is sent at a random time between 2pm and 6pm. Craving prompt 4 and Smoking prompt 2 are sent at a fixed time at 7pm. For each craving prompt, the participant responds to the item “Right now, how much are you craving a cigarette?” on a sliding scale from 0 = not at all to 100 = extremely in increments of one. For Smoking prompt 1, the participant responds to the item, “Between 7am and 1pm, how many cigarettes have you smoked in total?” using a numeric entry. For Smoking prompt 2, the participant responds to the item, “Between 1pm and 7pm, how many cigarettes have you smoked in total?” using a numeric entry. Hour-level smoking prompts are sent with craving prompts.

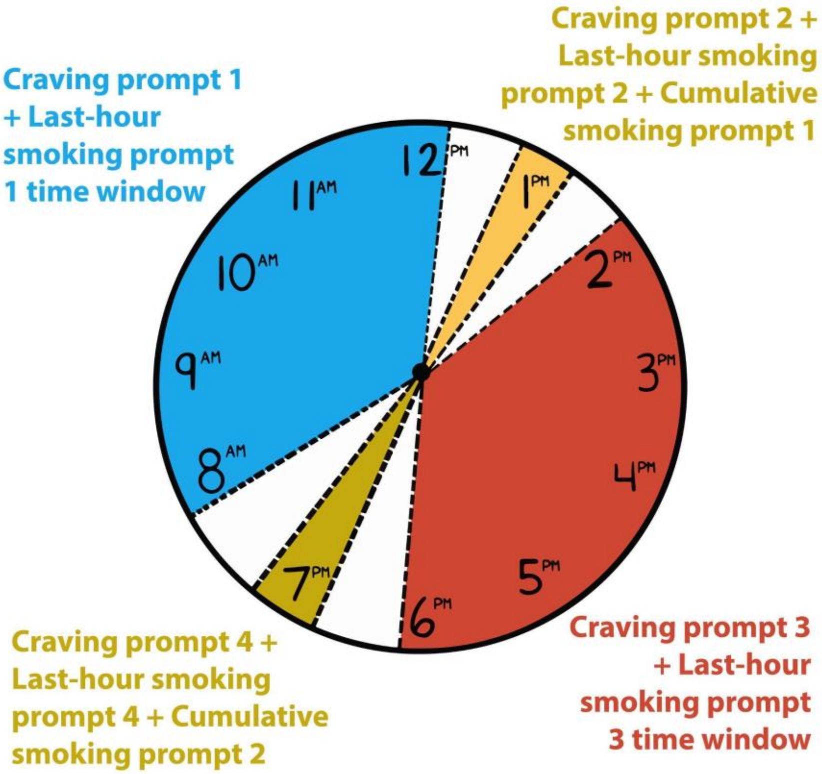

© 2025 Muzekari B et al. *JAMA Network Open*.

eFigure 3. Associations Between Within-Person Daily Tobacco Retail Exposure and Craving and Smoking, Including Smoking Outlier. Displayed are the primary day-level models including the smoking outlier 8.46 SD above the mean. The averaged fixed effect for within-person daily tobacco retail exposure is shown in red. Grey lines visualize individual-level associations between within-person tobacco retail exposure and craving and daily cigarettes smoked, allowing for both random intercepts and slopes. See eTables 1-13 for statistics for all model specifications.

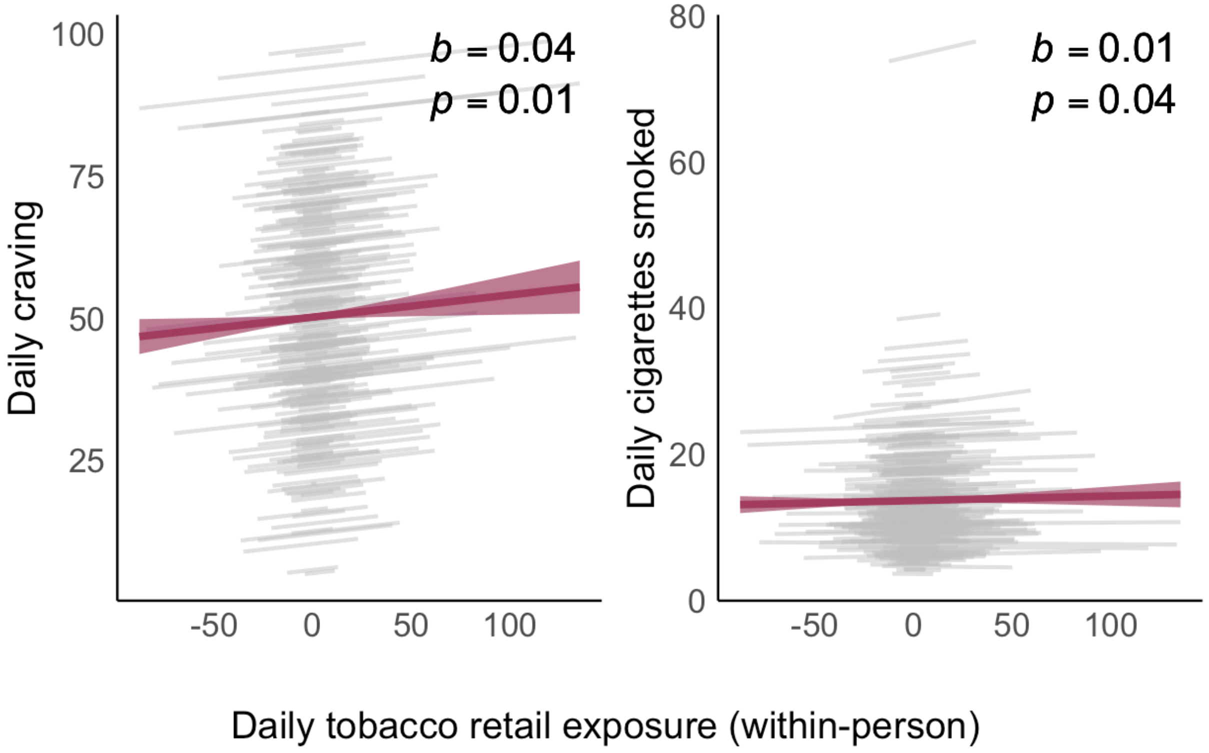

© 2025 Muzekari B et al. *JAMA Network Open*.

eFigure 4. Associations Between Within-Person Hour-Level Tobacco Retail Exposure and Craving and Smoking, Including Smoking Outlier. Displayed are the primary hour-level models including the smoking outlier 8.18 SD above the mean. The averaged fixed effect for within-person hourly tobacco retail exposure is shown in red. Grey lines visualize individual-level associations between within-person tobacco retail exposure and craving and cigarettes smoked in the past hour, allowing for both random intercepts and slopes. See eTables 14-18 for statistics for all model specifications.

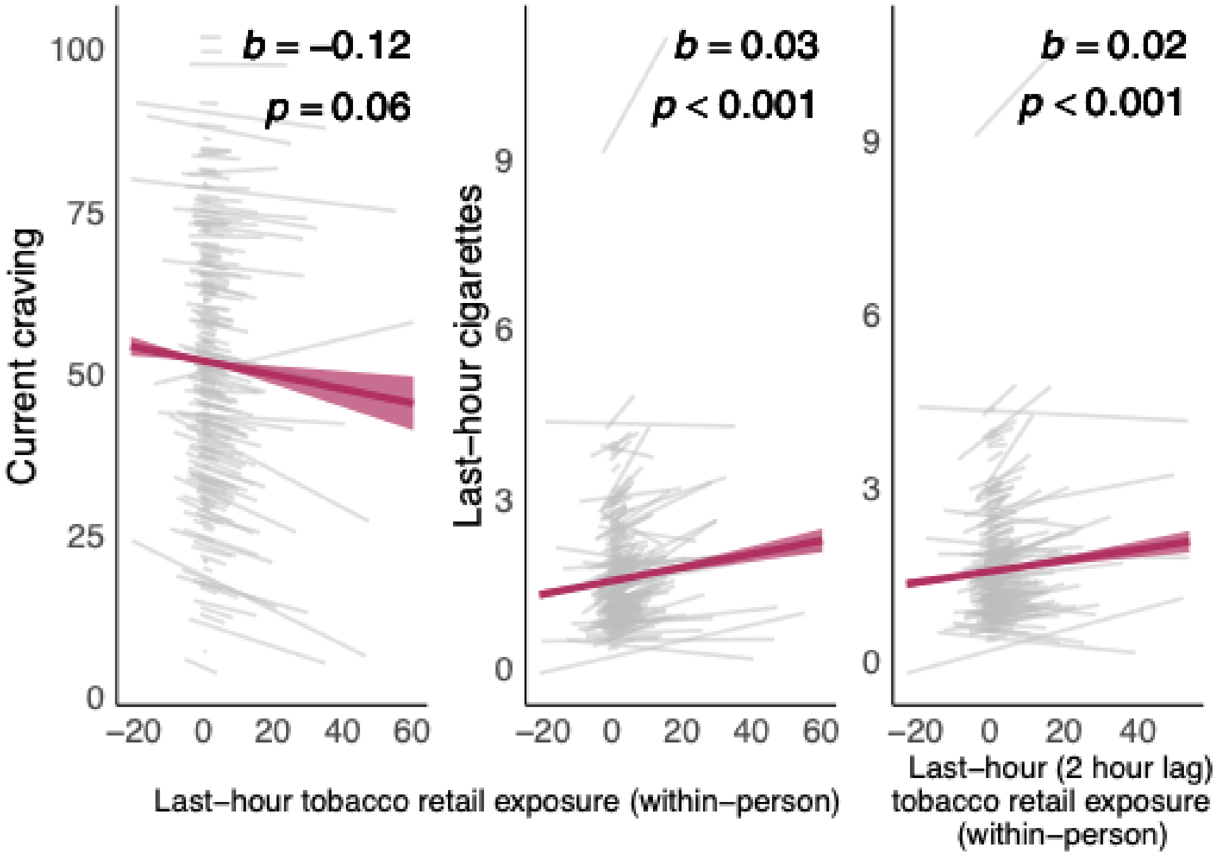

© 2025 Muzekari B et al. *JAMA Network Open*.

eTable 1. Daily Craving (Winsorized): Day Level

	Daily craving (winsorized)				
**Effect**	**Estimate**	**SE**	***t*-value**	***p*-value**	**95% CI**
Intercept	50.17	1.35	37.19	<0.001	[47.53,52.82]
Within-person daily exposure	0.04	0.01	2.72	0.01	[0.01,0.07]
Between-person daily exposure	0.03	0.04	0.80	0.43	[-0.05,0.11]
Day	-0.23	0.07	-3.10	0.002	[-0.37,-0.08]
σ^2	248.14				
τ_{00}	388.37				
ICC	0.61				
N	273				
Observations	3,732				
Marginal R^2/Conditional R^2	0.003/0.61				

© 2025 Muzekari B et al. *JAMA Network Open*.

eTable 2. Daily Craving (Nonwinsorized): Day Level

	Daily craving (non-winsorized)				
**Effect**	**Estimate**	**SE**	***t*-value**	***p*-value**	**95% CI**
Intercept	50.34	1.35	37.16	<0.001	[47.69,53]
Within-person daily exposure	0.04	0.01	2.71	0.01	[0.01,0.07]
Between-person daily exposure	0.03	0.04	0.86	0.39	[-0.04,0.11]
Day	-0.25	0.08	-3.15	0.001	[-0.41,-0.10]
σ^2	293.77				
τ_{00}	374.52				
ICC	0.56				
N	273				
Observations	3,732				
Marginal R^2/Conditional R^2	0.004/0.56				

© 2025 Muzekari B et al. *JAMA Network Open*.

eTable 3. Daily Cigarettes (Winsorized): Day Level

	Daily cigarettes (winsorized)				
**Effect**	**Estimate**	**SE**	***t*-value**	***p*-value**	**95% CI**
Intercept	13.63	0.46	29.57	<0.001	[12.73,14.54]
Within-person daily exposure	0.01	0.003	2.05	0.04	[<0.001,0.01]
Between-person daily exposure	0.01	0.01	0.52	0.60	[-0.02,0.04]
Day	-0.01	0.02	-0.72	0.47	[-0.04,0.02]
σ^2	11.38				
τ_{00}	52.65				
ICC	0.82				
N	273				
Observations	3,744				
Marginal R^2/Conditional R^2	0.001/0.82				

© 2025 Muzekari B et al. *JAMA Network Open*.

eTable 4. Daily Cigarettes (Nonwinsorized): Day Level. Due to failed convergence, only a random intercept was specified in this model.

	**Daily cigarettes (non-winsorized)**				
**Effect**	**Estimate**	**SE**	***t*-value**	***p*-value**	**95% CI**
Intercept	13.69	0.47	29.37	<0.001	[12.78,14.61]
Within-person daily exposure	0.01	0.003	2.26	0.02	[<0.001,0.01]
Between-person daily exposure	0.01	0.01	0.46	0.64	[-0.02,0.04]
Day	-0.01	0.02	-0.78	0.44	[-0.05,0.02]
σ^2	15.22				
τ_{00}	52.53				
ICC	0.78				
N	273				
Observations	3,744				
Marginal R^2/Conditional R^2	0.0009/0.78				

© 2025 Muzekari B et al. *JAMA Network Open*.

eTable 5. Daily Cigarettes (Excluding Smoking Outlier 8.46 SD Above the Mean): Day Level

	Daily cigarettes (excluding outlier)				
**Effect**	**Estimate**	**SE**	***t*-value**	***p*-value**	**95% CI**
Intercept	13.40	0.40	33.41	<0.001	[12.62,14.19]
Within-person daily exposure	0.01	0.003	2.01	0.04	[<0.001,0.01]
Between-person daily exposure	0.01	0.01	0.84	0.40	[-0.01,0.04]
Day	-0.01	0.02	-0.69	0.49	[-0.04,0.02]
σ^2	11.33				
τ_{00}	38.39				
ICC	0.77				
N	272				
Observations	3,730				
Marginal R^2/Conditional R^2	0.002/0.77				

© 2025 Muzekari B et al. *JAMA Network Open*.

eTable 6. Daily Craving and Cigarettes (Age as a Covariate): Day Level

	Daily craving					Daily cigarettes				
**Effect**	**Estimate**	**SE**	***t*-value**	***p*-value**	**95% CI**	**Estimate**	**SE**	***t*-value**	***p*-value**	**95% CI**
Intercept	68.71	5	13.76	<0.001	[58.92,78.5]	10.23	1.82	5.61	<0.001	[6.65, 13.8]
Age	-0.44	0.11	-3.85	<0.001	[-0.66,-0.21]	0.08	0.04	1.93	0.05	[-0.001, 0.16]
Within-person daily exposure	0.04	0.01	2.72	0.01	[0.01, 0.07]	0.01	0.003	2.02	0.04	[<0.001,0.01]
Between-person daily exposure	0.05	0.04	1.23	0.22	[-0.03,0.13]	0.004	0.01	0.31	0.76	[-0.02,0.03]
Day	-0.23	0.07	-3.09	0.002	[-0.37,-0.08]	-0.01	0.02	-0.72	0.47	[-0.04,0.02]
σ^2	248.15					11.38				
τ_{00}	368.10					52.04				
ICC	0.60					0.82				
N	273					273				
Observations	3,732					3,744				
Marginal R^2/Conditional R^2	0.04/0.61					0.82				

© 2025 Muzekari B et al. *JAMA Network Open*.

eTable 7. Daily Craving and Cigarettes (Gender as a Covariate): Day Level

	Daily craving					Daily cigarettes				
**Effect**	**Estimate**	**SE**	***t*-value**	***p*-value**	**95% CI**	**Estimate**	**SE**	***t*-value**	***p*-value**	**95% CI**
Intercept	48.21	1.76	27.44	<0.001	[44.76,51.65]	13.98	0.61	22.85	<0.001	[12.78,15.18]
Gender: Genderfluid	-13.30	20.45	-0.65	0.52	[-53.55, 26.96]	4.65	7.32	0.64	0.53	[-9.76,19.06]
Gender: Genderqueer	14.46	14.51	1	0.32	[-14.1,43.02]	6.2	5.2	1.19	0.23	[-4.05,16.44]
Gender: Non-binary	7.53	8.49	0.89	0.38	[-9.18,24.24]	-5.13	3.05	-1.68	0.09	[-11.13, 0.87]
Gender: Man	4.28	2.59	1.66	0.10	[-0.81,9.37]	-0.74	0.93	-0.79	0.43	[-2.56,1.09]
Within-person daily exposure	0.04	0.01	2.71	0.01	[0.01, 0.07]	0.01	0.003	2.08	0.04	[<0.001,0.01]
Between-person daily exposure	0.02	0.04	0.58	0.56	[-0.06,0.11]	0.01	0.01	0.58	0.56	[-0.02,0.04]
Day	-0.23	0.07	-3.15	0.001	[-0.38,-0.09	-0.01	0.02	-0.72	0.47	[-0.04,0.02]
σ^2	247.85					11.37				
τ_{00}	390.33					52.54				
ICC	0.61					0.82				
N	271					271				
Observations	3,705					3,716				
Marginal R^2/Conditional R^2	0.01/0.62					0.02/0.83				

© 2025 Muzekari B et al. *JAMA Network Open*.

eTable 8. Daily Craving And Cigarettes (Race as a Covariate): Day Level

	Daily craving					Daily cigarettes				
**Effect**	**Estimate**	**SE**	***t*-value**	***p*-value**	**95% CI**	**Estimate**	**SE**	***t*-value**	***p*-value**	**95% CI**
Intercept	49.68	1.63	30.56	<0.001	[46.49,52.87]	14.84	0.52	26.64	<0.001	[13.75,15.93]
Race: American Indian or Alaska Native	13.24	14.37	0.92	0.36	[-15.06,41.54]	-0.47	5.11	-0.09	0.93	[-10.52,9.59]
Race: Asian	4.11	7.78	0.53	0.6	[-11.2,19.43]	-4.00	2.76	-1.45	0.15	[-9.45,1.44]
Race: Black	-2.05	2.96	-0.69	0.49	[-7.87,3.77]	-4.21	1.05	-4.02	<0.001	[-6.27,-2.15]
Race: More than one	1.39	5.87	0.24	0.81	[-10.18,12.96]	-2.06	2.08	-0.99	0.32	[-6.14,2.03]
Race: Prefer not to say	19.16	20.21	0.95	0.34	[-20.64,58.96]	9.01	7.16	1.26	0.21	[-5.08,23.10]
Race: Prefer to self-describe	16.02	7.29	2.2	0.03	[1.67,30.37]	-0.80	2.59	-0.31	0.76	[-5.89,4.3]
Within-person daily exposure	0.04	0.01	2.71	0.01	[0.01,0.07]	0.01	0.003	2.15	0.03	[<0.001,0.01]
Between-person daily exposure	0.02	0.04	0.39	0.7	[-0.07,0.1]	0.02	0.02	1.03	0.30	[-0.01,0.05]
Day	-0.24	0.07	-3.16	0.001	[-0.38,-0.09]	-0.01	0.02	-0.64	0.52	[-0.04,0.02]
σ^2	248.74					11.38				
τ_{00}	381.27					50.23				
ICC	0.61					0.82				
N	270					270				
Observations	3,691					3,702				
Marginal R^2/Conditional R^2	0.02/0.61					0.06/0.83				

© 2025 Muzekari B et al. *JAMA Network Open*.

eTable 9. Daily Craving and Cigarettes (Ethnicity as a Covariate): Day Level

	Daily craving					Daily cigarettes				
**Effect**	**Estimate**	**SE**	***t*-value**	***p*-value**	**95% CI**	**Estimate**	**SE**	***t*-value**	***p*-value**	**95% CI**
Intercept	49.27	1.38	35.67	<0.001	[46.57,51.98]	13.87	0.48	29.06	<0.001	[12.94,14.81]
Ethnicity: Hispanic or Latinx	14.78	4.91	3.01	0.002	[5.11, 24.44]	-3.23	1.78	-1.82	0.07	[-6.74,0.27]
Ethnicity: Prefer not to say	-17.74	20.16	-0.88	0.38	[-57.44, 21.95]	-8.70	7.26	-1.2	0.23	[-23,5.59]
Within-person daily exposure	0.04	0.01	2.71	0.01	[0.01, 0.07]	0.01	0.003	2.08	0.04	[<0.001,0.01]
Between-person daily exposure	0.04	0.04	0.87	0.38	[-0.04,0.12]	0.01	0.01	0.57	0.57	[-0.02,0.04]
Day	-0.23	0.07	-3.16	0.001	[-0.38,-0.09]	-0.01	0.02	-0.73	0.47	[-0.04,0.02]
σ^2	247.86					11.37				
τ_{00}	379.15					52.37				
ICC	0.6					0.82				
N	271					271				
Observations	3,705					3,716				
Marginal R^2/Conditional R^2	0.03/0.62					0.02/0.82				

© 2025 Muzekari B et al. *JAMA Network Open*.

eTable 10. Daily Craving and Cigarettes (Fagerström Test for Nicotine Dependence as a Covariate): Day Level

	Daily craving					Daily cigarettes				
**Effect**	**Estimate**	**SE**	***t*-value**	***p*-value**	**95% CI**	**Estimate**	**SE**	***t*-value**	***p*-value**	**95% CI**
Intercept	41.66	3.83	10.89	<0.001	[34.16,49.16]	4.89	1.26	3.89	<0.001	[2.42, 7.35]
Fagerström Test for Nicotine Dependence	1.52	0.64	2.38	0.02	[0.26, 2.77]	1.56	0.21	7.43	<0.001	[1.15, 1.97]
Within-person daily exposure	0.04	0.01	2.77	0.01	[0.01, 0.07]	0.01	0.003	2.15	0.03	[<0.001,0.01]
Between-person daily exposure	0.03	0.04	0.71	0.48	[-0.05,0.11]	0.002	0.01	0.16	0.87	[-0.02,0.03]
Day	-0.22	0.07	-3.03	0.002	[-0.37,-0.08]	-0.01	0.02	-0.64	0.52	[-0.04,0.02]
σ^2	247.83					11.41				
τ_{00}	384.42					43.68				
ICC	0.61					0.79				
N	270					270				
Observations	3,691					3,702				
Marginal R^2/Conditional R^2	0.02/0.61					0.14/0.82				

© 2025 Muzekari B et al. *JAMA Network Open*.

eTable 11. Daily Craving and Cigarettes (Smartphone Type as a Covariate): Day Level

	**Daily craving**					**Daily cigarettes**				
**Effect**	**Estimate**	**SE**	***t*-value**	***p*-value**	**95% CI**	**Estimate**	**SE**	***t*-value**	***p*-value**	**95% CI**
Intercept	49	1.9	25.74	<0.001	[45.27,52.73]	13.71	0.67	20.5	<0.001	[12.4, 15.01]
Smartphone type: IOS	2.66	3.05	0.87	0.38	[-3.34,8.66]	-0.16	1.09	-0.15	0.88	[-2.32,1.99]
Within-person daily exposure	0.04	0.01	2.72	0.01	[0.01, 0.07]	0.01	0.003	2.04	0.04	[<0.001,0.01]
Between-person daily exposure	0.06	0.05	1.16	0.25	[-0.04,0.16]	0.01	0.02	0.34	0.74	[-0.03,0.04]
Day	-0.23	0.07	-3.09	0.002	[-0.37,0.08]	-0.01	0.02	-0.72	0.47	[-0.04,0.02]
σ^2	248.14					11.38				
τ_{00}	388.75					52.85				
ICC	0.61					0.82				
N	273					273				
Observations	3,732					0.001				
Marginal R^2/Conditional R^2	0.005/0.61					0.82				

© 2025 Muzekari B et al. *JAMA Network Open*.

eTable 12. Daily Craving and Cigarettes (State as a Covariate): Day Level

	Daily craving					Daily cigarettes				
**Effect**	**Estimate**	**SE**	***t*-value**	***p*-value**	**95% CI**	**Estimate**	**SE**	***t*-value**	***p*-value**	**95% CI**
Intercept	49.66	1.53	32.52	<0.001	[46.67,52.65]	13.46	0.52	25.76	<0.001	[12.44,14.49]
State: Delaware	0.79	4.32	0.18	0.86	[-7.72,9.30]	3.43	1.54	2.23	0.03	[0.4,6.45]
State: New Jersey	2.78	3.42	0.81	0.42	[-3.96,9.52]	-0.89	1.22	-0.73	0.46	[-3.29,1.5]
Within-person daily exposure	0.04	0.01	2.72	0.01	[0.01, 0.07]	0.01	0.003	2.03	0.04	[<0.001,0.01]
Between-person daily exposure	0.03	0.04	0.81	0.41	[-0.05,0.11]	0.01	0.01	0.52	0.6	[-0.02,0.04]
Day	-0.23	0.07	-3.1	0.002	[-0.37,-0.08]	-0.01	0.02	-0.72	0.47	[-0.04,0.02]
σ^2	248.14					11.38				
τ_{00}	390.41					51.75				
ICC	0.611					0.82				
N	273					273				
Observations	3,732					3,744				
Marginal R^2/Conditional R^2	0.005/0.61					0.02/0.82				

© 2025 Muzekari B et al. *JAMA Network Open*.

eTable 13. Daily Craving and Cigarettes (All Covariates Included): Day Level

	Daily craving					Daily cigarettes				
**Effect**	**Estimate**	**SE**	***t*-value**	***p*-value**	**95% CI**	**Estimate**	**SE**	***t*-value**	***p*-value**	**95% CI**
Intercept	53.8	6.85	7.85	<0.001	[40.37,67.24]	4.72	2.31	2.04	0.04	[0.19, 9.25]
Age	-0.42	0.12	-3.42	<0.001	[-0.66,-0.18]	0.05	0.04	1.1	0.27	[0.04, 0.13]
Gender: Genderfluid	-28.72	21.36	-1.34	0.18	[-70.78, 13.34]	0.29	7.21	0.04	0.97	[-13.92, 14.49]
Gender: Genderqueer	11.33	14.25	0.8	0.43	[-16.74, 39.40]	4.53	4.82	0.94	0.35	[-4.96,14.01]
Gender: Man	2.66	2.61	1.02	0.31	[-2.48,7.8]	0.24	0.88	0.27	0.79	[-1.5,1.98]
Gender: Non-binary	3.01	8.54	0.35	0.73	[-13.82, 19.84]	-2.44	2.89	-0.85	0.4	[-8.13,3.25]
Race: American Indian or Alaska Native	3.97	14.34	0.28	0.78	[-24.28, 32.21]	0.33	4.84	0.07	0.95	[-9.21,9.87]
Race: Asian	2.28	7.72	0.3	0.77	[-12.92, 17.49]	-3.19	2.61	-1.22	0.22	[-8.33,1.96]
Race: Black	0.48	2.98	0.16	0.87	[-5.29,6.36]	-3.25	1.01	-3.22	0.001	[-5.23,-1.26]
Race: More than one	1.9	5.79	0.33	0.74	[-9.5,13.31]	-0.7	1.95	-0.36	0.72	[-4.55,3.14]
Race: Prefer not to say	11.39	19.83	0.57	0.57	[-27.67, 50.44]	7.67	6.69	1.15	0.25	[-5.51,20.85]
Race: Prefer to self-describe	12.6	8.19	1.54	0.13	[-3.53,28.74]	1.33	2.77	0.48	0.63	[-4.12,6.78]
Ethnicity: Hispanic or Latinx	7.23	5.51	0.33	0.74	[-3.61,18.08]	-2.44	1.86	-1.31	0.19	[-6.1,1.23]
Fagerström Test for Nicotine Dependence	2.03	0.66	3.08	0.002	[0.73, 3.33]	1.32	0.22	5.93	<0.001	[0.88, 1.76]
Smartphone type: IOS	0.19	3.12	0.06	0.95	[-5.96,6.33]	1.12	1.05	1.06	0.29	[0.96, 3.19]

© 2025 Muzekari B et al. *JAMA Network Open*.

State: Delaware	-0.34	4.34	-0.08	0.94	[-8.89,8.21]	1.51	1.47	1.03	0.31	[-1.38,4.4]
State: New Jersey	0.45	3.46	0.13	0.9	[-6.37,7.26]	-0.9	1.17	-0.77	0.44	[-3.2,1.4]
Within-person daily exposure	0.04	0.01	2.77	0.01	[0.01, 0.07]	0.01	0.003	2.21	0.03	[<0.001,0.01]
Between-person daily exposure	0.02	0.05	0.42	0.67	[-0.08,0.12]	0.02	0.02	0.99	0.32	[0.02, 0.05]
Day	-0.23	0.07	-3.03	0.002	[-0.37,-0.08	-0.01	0.02	-0.56	0.57	[-0.04,0.02]
σ^2	248.74					11.42				
τ_{00}	357.83					42.54				
ICC	0.59					0.79				
N	269					269				
Observations	3,677					3,688				
Marginal R^2/Conditional R^2	0.08/0.62					0.19/0.83				

© 2025 Muzekari B et al. *JAMA Network Open*.

eTable 14. Current Craving: Hour Level

	**Current craving (time-since-start terms included)**					**Current craving (no time-since-start terms)**				
**Effect**	**Estimate**	**SE**	***t*-value**	***p*-value**	**95% CI**	**Estimate**	**SE**	***t*-value**	***p*-value**	**95% CI**
Intercept	51.81	1.69	31.06	<0.001	[48.54,55.08]	49.86	1.32	37.71	<0.001	[47.27,52.45]
Time-since-start linear	0.01	0.01	0.67	0.51	[-0.01,0.02]					
Time-since-start quadratic	-0.00003	<0.0001	-2.22	0.03	[>-0.001, >-0.001,]					
Time-since-start cubic	<0.0001	<0.0001	2.83	0.004	[<0.001, <0.001]					
Within-person last-hour exposure	-0.12	0.06	-1.9	0.06	[-0.25,0.003]	-0.11	0.06	-1.75	0.08	[-0.24,0.31]
Between-person last-hour exposure	0.37	0.32	1.15	0.25	[-0.26,0.99]	0.37	0.32	1.16	0.25	[-0.26,1]
Day	-0.22	0.06	-3.46	<0.001	[-0.34,-0.1]	-0.22	0.06	-3.48	<0.001	[-0.35,-0.1]
σ^2	906.6					909.39				
τ_{00}	398.99					398.29				
ICC	0.31					0.3				
N	273					273				
Observations	14,182					14,182				
Marginal R^2/Conditional R^2	0.004/0.31					0.002/0.31				

© 2025 Muzekari B et al. *JAMA Network Open*.

eTable 15. Current Craving (Last-Hour Cigarettes as a Covariate): Hour-Level

	Current craving (time-since-start terms included)					Current craving (no time-since-start terms)				
**Effect**	**Estimate**	**SE**	***t*-value**	***p*-value**	**95% CI**	**Estimate**	**SE**	***t*-value**	***p*-value**	**95% CI**
Intercept	51.69	1.66	31.21	<0.001	[48.44,54.94]	50.26	1.32	38.1	<0.001	[47.68,52.85]
Within-person last-hour cigarettes	-6.2	0.27	-23.35	<0.001	[-6.72,-5.68]	-6.31	0.26	-23.81	<0.001	[-6.83,-5.79]
Time-since-start linear	0.003	0.01	0.44	0.66	[-0.01,0.02]					
Time-since-start quadratic	>-0.001	<0.001	-1.49	0.14	[>-0.001,<0.001,]					
Time-since-start cubic	<0.001	<0.001	1.81	0.07	[>-0.001,<0.001,]					
Within-person last-hour exposure	-0.03	0.07	-0.44	0.66	[-0.16,0.1]	-0.02	0.07	-0.26	0.8	[-0.15,0.11]
Between-person last-hour exposure	0.4	0.32	1.27	0.21	[-0.22,1.03]	0.4	0.32	1.27	0.2	[-0.22,1.03]
Day	-0.28	0.06	-4.42	<0.001	[-0.4,-0.15]	-0.28	0.06	-4.44	<0.001	[-0.4,-0.15]
σ^2	871.91					873.24				
τ_{00}	399.7					399.17				
ICC	0.31					0.31				
N	273					273				
Observations	14,129					14.129				
Marginal R^2/Conditional R^2	0.03/0.34					0.03/0.33				

© 2025 Muzekari B et al. *JAMA Network Open*.

eTable 16. Last-Hour Cigarettes: Hour Level

	Last-hour cigarettes (time-since-start terms included)					Last-hour cigarettes (no time-since-start terms)				
**Effect**	**Estimate**	**SE**	***t*-value**	***p*-value**	**95% CI**	**Estimate**	**SE**	***t*-value**	***p*-value**	**95% CI**
Intercept	1.53	0.07	22.39	<0.001	[1.40, 1.67]	1.61	0.06	26.6	<0.001	[1.49, 1.73]
Time-since-start linear	-0.0003	0.0002	-1.27	0.2	[>-0.001, >-0.001,]					
Time-since-start quadratic	<0.0001	<0.0001	3.87	<0.001	[<0.001, <0.001]					
Time-since-start cubic	<0.0001	<0.0001	-5.45	<0.001	[>-0.001, >-0.001,]					
Within-person last-hour exposure	0.03	0.003	6.65	<0.001	[0.02, 0.03]	0.02	0.003	6.65	<0.001	[0.02, 0.03]
Between-person last-hour exposure	0.02	0.01	1.47	0.14	[-0.01,0.05]	0.02	0.01	1.42	0.16	[-0.01,0.05]
Day	-0.01	0.001	-5.13	<0.001	[-0.01,-0.01]	-0.01	0.002	-5.04	<0.001	[-0.01,-0.01]
σ^2	0.88					0.89				
τ_{00}	0.93					0.93				
ICC	0.51					0.51				
N	273					273				
Observations	14,146					14,146				
Marginal R^2/Conditional R^2	0.02/0.53					0.01/0.52				

© 2025 Muzekari B et al. *JAMA Network Open*.

eTable 17. Last-Hour Cigarettes (2-Hour Exposure Lag): Hour Level

	Last-hour cigarettes (time-since-start terms included)					Last-hour cigarettes (no time-since-start terms)				
**Effect**	**Estimate**	**SE**	***t*-value**	***p*-value**	**95% CI**	**Estimate**	**SE**	***t*-value**	***p*-value**	**95% CI**
Intercept	1.53	0.07	22.35	<0.001	[1.4,1.66]	1.62	0.06	26.7	<0.001	[1.5,1.73]
Time-since-start linear	-0.0002	0.0002	-0.88	0.38	[>-0.001, >-0.001,]					
Time-since-start quadratic	<0.0001	<0.0001	3.45	<0.001	[<0.001, <0.001]					
Time-since-start cubic	<0.0001	<0.0001	-5.12	<0.001	[>-0.001, >-0.001,]					
Within-person last-hour exposure	0.02	0.003	4.31	<0.001	[0.01, 0.02]	0.02	0.003	4.49	<0.001	[0.01, 0.02]
Between-person last-hour exposure	0.02	0.02	1.4	0.16	[-0.01,0.05]	0.02	0.02	1.38	0.17	[-0.01,0.05]
Day	-0.01	0.001	-5.31	<0.001	[-0.01,-0.01]	-0.01	0.002	-5.21	<0.001	[-0.01,-0.01]
σ^2	0.89					0.89				
τ_{00}	0.92					0.92				
ICC	0.51					0.51				
N	273					273				
Observations	14,146					14,146				
Marginal R^2/Conditional R^2	0.01/0.52					0.01/0.52				

© 2025 Muzekari B et al. *JAMA Network Open*.

eTable 18. Last-Hour Cigarettes (Excluding Smoking Outlier 8.18 SD Above the Mean): Hour Level (1-Hour and 2-Hour Exposure Lag)

	Last-hour cigarettes (1-Hour Lag)					Last-hour cigarettes (2-Hour Lag)				
**Effect**	**Estimate**	**SE**	***t*-value**	***p*-value**	**95% CI**	**Estimate**	**SE**	***t*-value**	***p*-value**	**95% CI**
Intercept	1.52	0.06	24.78	<0.001	[1.4,1.64]	1.52	0.06	24.68	<0.001	[1.4,1.64]
Time-since-start linear	-0.0004	0.0002	-1.77	0.08	[>-0.001,<0.001,]	-0.0003	0.0002	-1.32	0.19	[>-0.001,<0.001,]
Time-since-start quadratic	<0.0001	<0.0001	4.34	<0.001	[<0.001,<0.001]	<0.0001	<0.0001	3.88	<0.001	[<0.001,<0.001]
Time-since-start cubic	<0.0001	<0.0001	-5.9	<0.001	[>-0.001,>-0.001,]	<0.0001	<0.0001	-5.52	<0.001	[>-0.001,>-0.001,]
Within-person last-hour exposure	0.03	0.003	6.63	<0.001	[0.02,0.03]	0.02	0.003	4.19	<0.001	[0.01,0.02]
Between-person last-hour exposure	0.02	0.01	1.35	0.18	[-0.01,0.04]	0.02	0.01	1.37	0.17	[-0.01,0.04]
Day	-0.01	0.001	-5.39	<0.001	[-0.01,-0.01]	-0.01	0.001	-5.58	<0.001	[-0.01,-0.01]
σ^2	0.84					0.84				
τ_{00}	0.69					0.69				
ICC	0.45					0.45				
N	272					272				
Observations	14,091					14.091				
Marginal R^2/Conditional R^2	0.02/0.47					0.01/0.46				

© 2025 Muzekari B et al. *JAMA Network Open*.

eTable 19. Craving and Smoking Association: Hour Level

	**Current craving (time-since-start terms included)**					**Current craving (no time-since-start terms)**				
**Effect**	**Estimate**	**SE**	***t*-value**	***p*-value**	**95% CI**	**Estimate**	**SE**	***t*-value**	***p*-value**	**95% CI**
Intercept	52.97	1.60	33.0	<0.001	[49.82,56.11]	50.49	1.28	39.36	<0.001	[47.98,53.0]
Time-since-start linear	-0.01	0.01	-0.72	0.47	[-0.02,0.01]					
Time-since-start quadratic	0.0	0.0	-0.29	0.77	[>-0.001, >-0.001]					
Time-since-start cubic	0.0	0.0	0.51	0.61	[>-0.001, >-0.001]					
Within-person last-hour cigarettes	-10.17	0.81	-12.5	<0.001	[-11.76, -8.57]	-10.24	0.81	-12.62	<0.001	[-11.83, -8.65]
Between-person last-hour cigarettes	4.64	1.24	3.74	<0.001	[2.2,7.09]	4.68	1.24	3.77	0.002	[2.24, 7.13]
Day	-0.31	0.06	-5.09	<0.001	[-0.42,-0.19]	-0.31	0.06	-5.09	<0.001	[-0.42,-0.19]
σ^2	786.83					788.38				
τ_{00}	379.81					379.13				
ICC	0.33					0.32				
N	273					273				
Observations	14,129					14,129				
Marginal R^2/Conditional R^2	0.08/0.44					0.08/0.44				

© 2025 Muzekari B et al. *JAMA Network Open*.

eTable 20. Current Craving and Next-Hour Exposure: Hour-Level Reverse

	Next-hour exposure (time-since-start terms included)					Next-hour exposure (no time-since-start terms)				
**Effect**	**Estimate**	**SE**	***t*-value**	***p*-value**	**95% CI**	**Estimate**	**SE**	***t*-value**	***p*-value**	**95% CI**
Intercept	2.2	0.28	7.83	<0.001	[1.65, 2.75]	2.51	0.24	10.42	<0.001	[2.04, 2.99]
Time-since-start linear	0.01	0.001	4.99	<0.001	[0.003,0.01]					
Time-since-start quadratic	>-0.0001	<0.0001	-5.1	<0.001	[>-0.001, >-0.001]					
Time-since-start cubic	0.0	0.0	3.08	0.002	[<0.001, <0.001]					
Within-person current craving	0.002	0.001	1.61	0.11	[>-0.001, 0.004]	0.002	0.001	1.95	0.05	[>-0.001, 0.01]
Between-person current craving	0.12	0.01	1.17	0.24	[-0.01,0.03]	0.01	0.01	1.22	0.22	[-0.01,0.03]
Day	0.003	0.01	0.35	0.73	[-0.01,0.02]	0.004	0.01	0.48	0.63	[-0.01,0.02]
σ^2	17.92					18.12				
τ_{00}	14.35					14.3				
ICC	0.44					0.44				
N	273					273				
Observations	14,182					14,182				
Marginal R^2/Conditional R^2	0.01/0.45					0.002/0.44				

© 2025 Muzekari B et al. *JAMA Network Open*.

eTable 21. Last-Hour Cigarettes and Next-Hour Exposure: Hour-Level Reverse

	Next-hour exposure (time-since-start terms included)					Next-hour exposure (no time-since-start terms)				
**Effect**	**Estimate**	**SE**	***t*-value**	***p*-value**	**95% CI**	**Estimate**	**SE**	***t*-value**	***p*-value**	**95% CI**
Intercept	2.18	0.28	7.77	<0.001	[1.63, 2.74]	2.51	0.24	10.37	<0.001	[2.03, 2.98]
Time-since-start linear	0.01	0.001	5.16	<0.001	[0.003,0.01]					
Time-since-start quadratic	>-0.0001	<0.0001	-5.36	<0.001	[>-0.001, >-0.001]					
Time-since-start cubic	0.0	0.0	3.38	<0.001	[<0.001, <0.001]					
Within-person last-hour cigarettes	0.11	0.05	2.32	0.02	[0.02, 0.20]	0.1	0.05	2.08	0.04	[0.01, 0.19]
Between-person last-hour cigarettes	0.25	0.2	1.22	0.23	[-0.15,0.65]	0.24	0.2	1.18	0.24	[-0.16,0.64]
Day	0.003	0.01	0.39	0.7	[-0.01,0.02]	0.004	0.01	0.5	0.61	[-0.01,0.22]
σ^2	17.76					17.97				
τ_{00}	14.44					0.45				
ICC	0.45					0.44				
N	273					273				
Observations	14,146					14,146				
Marginal R^2/Conditional R^2	0.01/0.46					0.001/0.45				

© 2025 Muzekari B et al. *JAMA Network Open*.
