# Supplementary material for: Naturalistic Tobacco Retail Exposure and Smoking Outcomes in Adults Who Smoke Cigarettes Daily
Source: JAMA Netw Open. 2025 Sep 29;8(9):e2530132. doi: 10.1001/jamanetworkopen.2025.30132 (PMC12481226; doi:10.1001/jamanetworkopen.2025.30132)
Supplement: Supplement 2. — Data Sharing Statement [file jamanetwopen-e2530132-s002.pdf]

Data Sharing Statement

Muzekari. Naturalistic Tobacco Retail Exposure and Smoking Outcomes in Adults Who Smoke Cigarettes Daily. *JAMA Netw Open*. Published September 29, 2025. doi:10.1001/jamanetworkopen.2025.30132

Data
Data available: No

Additional Information
Explanation for why data not available: Final research data, with all identity-related information deleted and in consultation with the relevant IRBs, will be made available to the scientific community for collaborative research with members of the study team, upon request, in spreadsheet format for all non-imaging data and in NIFTI format for fMRI data. Qualified investigators who wish to access the study materials will be able to complete a form https://docs.google.com/forms/d/1fn5fvi06AI6AKEfLupUeO-40WezRAaKCqyGUTHbLFhc/viewform?edit_requested=true. The requests will be reviewed and approved by the investigators in consultation with the relevant IRBs. Data that may be difficult to de-identify (including geolocation data) will be shared at an aggregate level, with restrictions as developed in consultation with the IRB, to protect participant privacy.
